# Supplementary material for: Complex‐centric proteome profiling by SEC‐SWATH‐MS
Source: Mol Syst Biol. 2019 Jan 14;15(1):e8438. doi: 10.15252/msb.20188438 (PMC6346213; doi:10.15252/msb.20188438)
Supplement: Supplementary file 8 — Dataset EV7 [file MSB-15-e8438-s008.zip › feature_plots_string/Q14563.pdf]

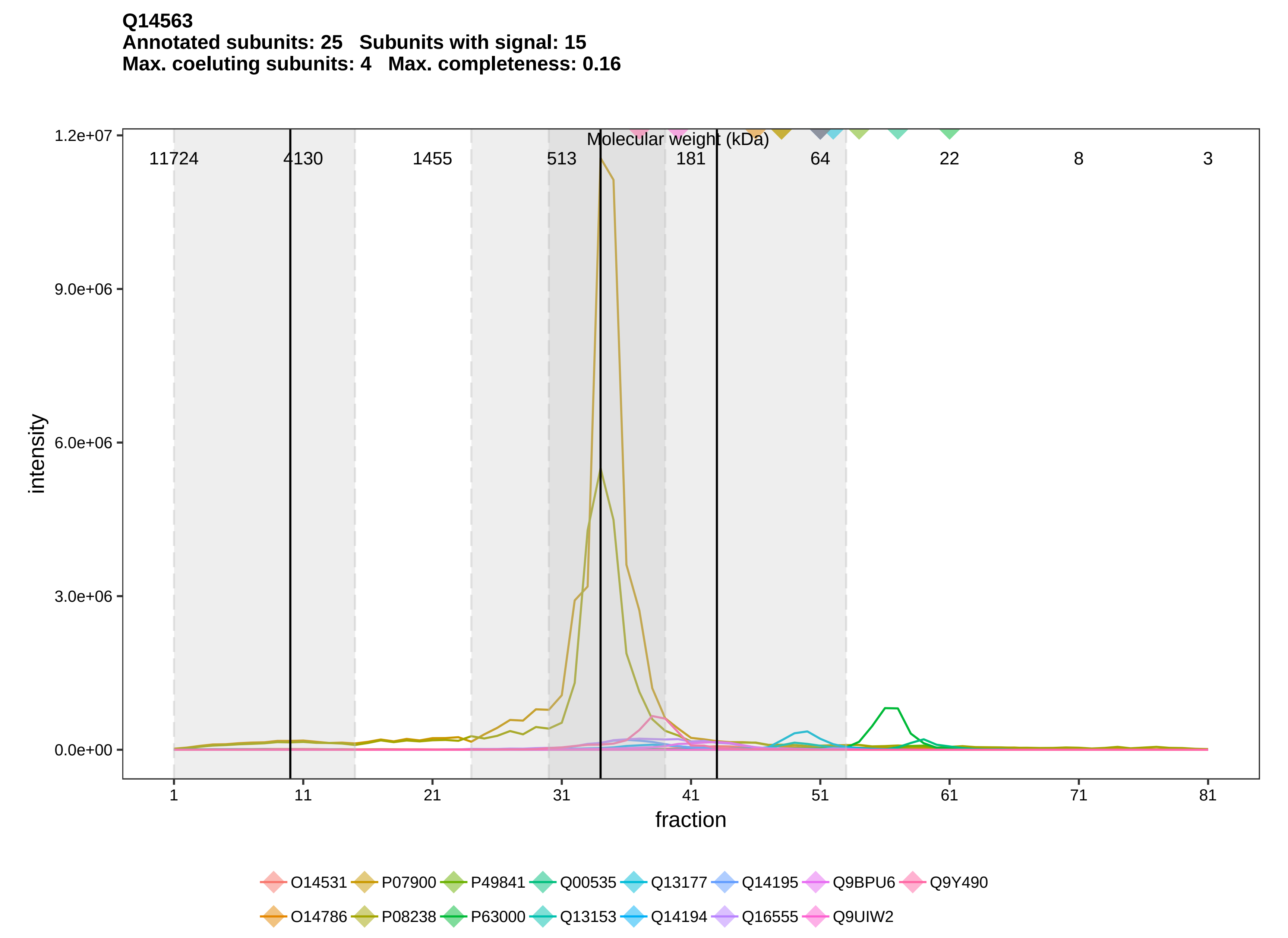
Q14563
Annotated subunits: 25 Subunits with signal: 15
Max. coeluting subunits: 4 Max. completeness: 0.16
Molecular weight (kDa)
11724
4130
1455
513
181
64
22
8
3
1.2e+07
9.0e+06
6.0e+06
3.0e+06
0.0e+00
intensity
1
11
21
31
41
51
61
71
81
fraction
O14531
P07900
P49841
Q00535
Q13177
Q14195
Q9BPU6
Q9Y490
O14786
P08238
P63000
Q13153
Q14194
Q16555
Q9UIW2
